# Distal chevron osteotomy versus different operative procedures for hallux valgus correction: a meta-analysis

**DOI:** 10.1186/s13018-022-02974-0

**Published:** 2022-02-08

**Authors:** Pablo Clemente, Gonzalo Mariscal, Carlos Barrios

**Affiliations:** 1 Department of Traumatology and Orthopedic Surgery, Hospital Doctor Peset, Valencia, Spain; 2 Institute for Research on Musculoskeletal Disorders, School of Medicine and Health Sciences, Valencia Catholic University, Quevedo 2, 46001 Valencia, Spain

**Keywords:** Chevron osteotomy, Hallux valgus, Distal chevron, Meta-analysis

## Abstract

**Introduction:**

Distal chevron osteotomy is commonly used for the operative treatment of hallux valgus (HV). However, there are several operative procedures that can be used to treat HV. The aim of this meta-analysis was to compare the efficacy of distal chevron osteotomy with different operative procedures.

**Materials and methods:**

A systematic search was conducted using the MEDLINE and EMBASE databases to identify randomized clinical trials (RCTs). The variables were radiological (hallux metatarsal phalangeal angle [HVA] and intermetatarsal angle [IMA]) and clinical (American Orthopaedic Foot & Ankle Society Score [AOFAS]). Heterogeneity was assessed with chi^2^ and *I*^2^ statistics. A random effects model was used for significant heterogeneity. Publication bias was evaluated with funnel plots.

**Results:**

Ten studies involving 985 patients were evaluated in the meta-analysis. Distal chevron osteotomy was associated with a mean IMA correction 2.18° greater than the scarf procedure (MD − 2.18; 95% CI − 3.67, − 0.69; *p* = 0.004; *I*^2^ = 0%). In addition, the proximal chevron was associated with a mean IMA correction 1.08° greater than the distal chevron (MD − 1.08; 95% CI − 1.86, − 0.29; *p* = 0.007; *I*^2^ = 0%). The AOFAS assessment showed an overall advantage of 3.2 points in favor of the Lingdren group compared with distal chevron osteotomy (MD 3.20; 95% CI 0.37, 6.04; *p* = 0.03; *I*^2^ = 0%).

**Conclusions:**

Our findings indicate that distal chevron osteotomy provides a greater HVA correction than scarf osteotomy, and proximal chevron provides a larger IMA correction than distal chevron osteotomy. Lingdren osteotomy provides a greater AOFAS correction than distal chevron osteotomy.

**Level of evidence:**

Level I, meta-analysis.

## Introduction

Hallux valgus (HV) is the most common forefoot pathology in adults. There is also a family history, with a higher prevalence in females [1]. HV is a complex progressive triplanar deformity of the forefoot, characterized by valgus phalangeal deviation, varus angulation of the first metatarsal, and lateral displacement of the sesamoids and extensor tendons. The initial sign is the formation of a medial bony prominence (bunionette) at the first metatarsophalangeal joint (MTP) [2]. The factors associated with the development of HV are multiple, but still unclear.

Mann and Coughlin classified HV into three types according to the hallux valgus angle (HVA) and the intermetatarsal angle (IMA): mild (HV < 20°, IMA < 11°), moderate (HV 20°–40°, IMA 11°–16°) and severe (HV > 40°, IMA > 16°) [3].

Several operative procedures have been described for the correction of HV. Conventionally, proximal and distal metatarsal osteotomies are used for severe or mild-moderate deformities, respectively [3, 4]. Distal chevron osteotomy alone or combined with other procedures has also shown improved radiological results and patient satisfaction [5]. The most serious adverse event associated with distal chevron osteotomy was osteonecrosis due to disruption of the blood supply to the metatarsal head. However, most studies supporting increased correction and functionality with distal chevron osteotomy have low evidence (level IV) [4]. Distal chevron osteotomy is a commonly used method for the correction of HV.^7^ There are several studies that compare it with other surgical techniques in isolated form [5–14].

The objective of this meta-analysis was to evaluate the optimal operative approach in hallux valgus. This meta-analysis examined different surgical options, comparing the clinical and radiological efficacy of the distal chevron osteotomy with other operative procedures, and thereby providing level I evidence for the surgical treatment of HV.

## Material and methods

### Search strategy and study selection

**Fig. 1:**
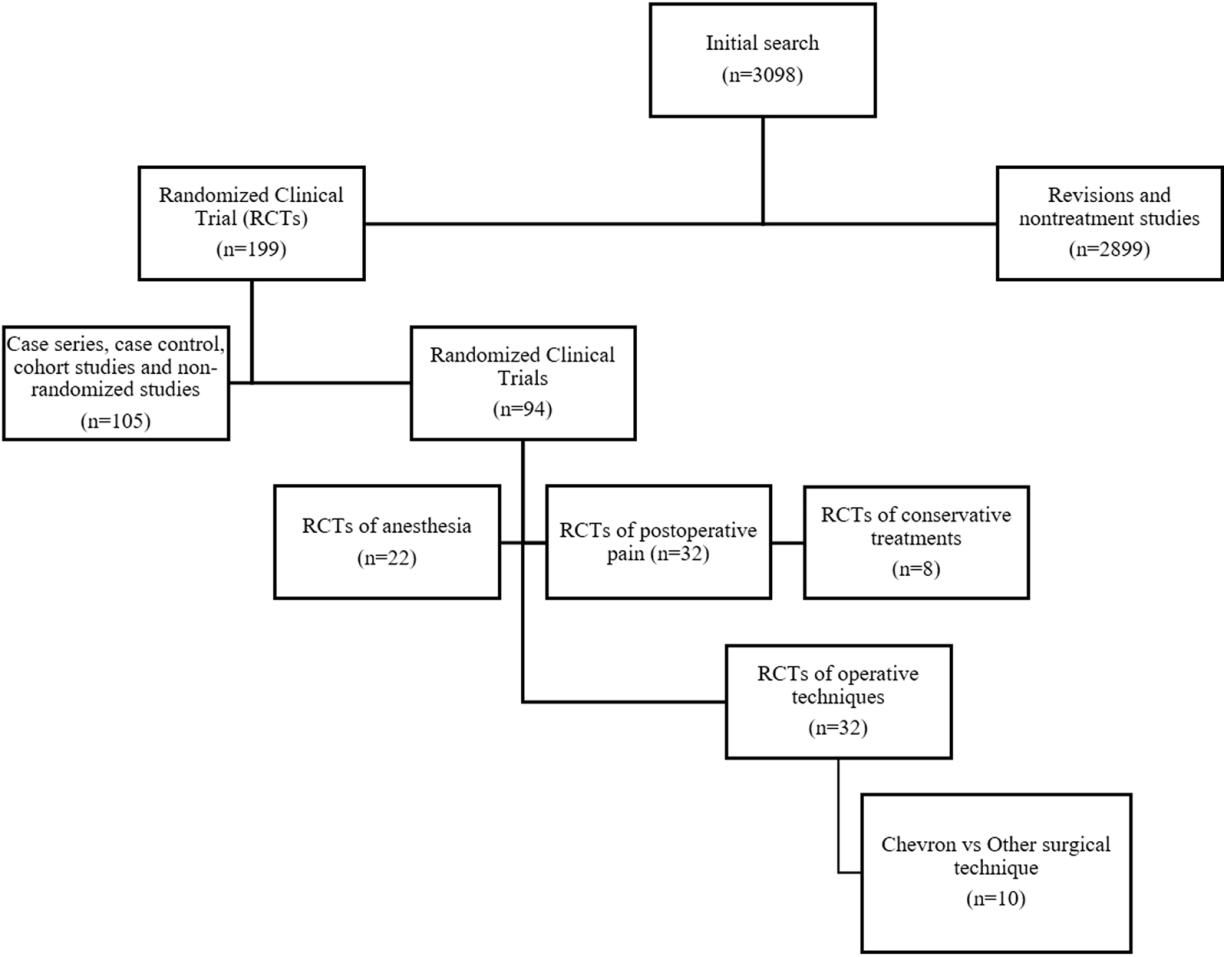
Study selection flow diagram (Preferred Reporting Items for Systematic Reviews and Meta-Analysis): The PRISMA diagram showing the exclusion and inclusion strategy of randomized clinical trials on distal chevron osteotomy of hallux valgus

The meta-analysis was carried out following the PRISMA criteria (Preferred Reporting Items for Systematic Reviews and Meta-Analyses) [15] (Fig. 1). A bibliographic search was carried out in PubMed, EMBASE, Scopus, and the Cochrane Collaboration Library. The following search terms were used: (1) hallux abducto valgus; (2) randomized clinical trial; and (3) treatment. The search was limited to studies published in the English language. The inclusion criteria were (1) randomized trials in the English language comparing operative techniques in patients with HV; (2) use of the distal chevron osteotomy in one of the study arms; and (3) posttreatment HVA and IMA data and AOFAS scores as the main outcome variables in each study. If more than one study reported data for the same sample, only the most recent and complete one was evaluated.

The results of the selection process are shown in Fig. 1. A total of 94 studies classified as randomized clinical trials on operative techniques were identified using the selected databases. After the first screening, 32 randomized clinical trials related to operative treatment were observed. Sixty-two studies were excluded because they were related to preoperative anesthesia (*n* = 22) postoperative pain control (*n* = 32), or conservative treatments (*n* = 8). Finally, 10 studies comparing chevron distal osteotomies with other surgical techniques were selected.

### Data extraction

The following baseline data were collected: intervention, number of patients, % of men, mean age, and follow-up (months). The study variables were divided in two groups: clinical parameters (American Orthopedic Foot and Ankle Score, AOFAS) and radiological measurements (hallux metatarsal phalangeal angle [HVA] and intermetatarsal angle [IMA] between the first and second radius).

### Assessment of study quality

**Fig. 2:**
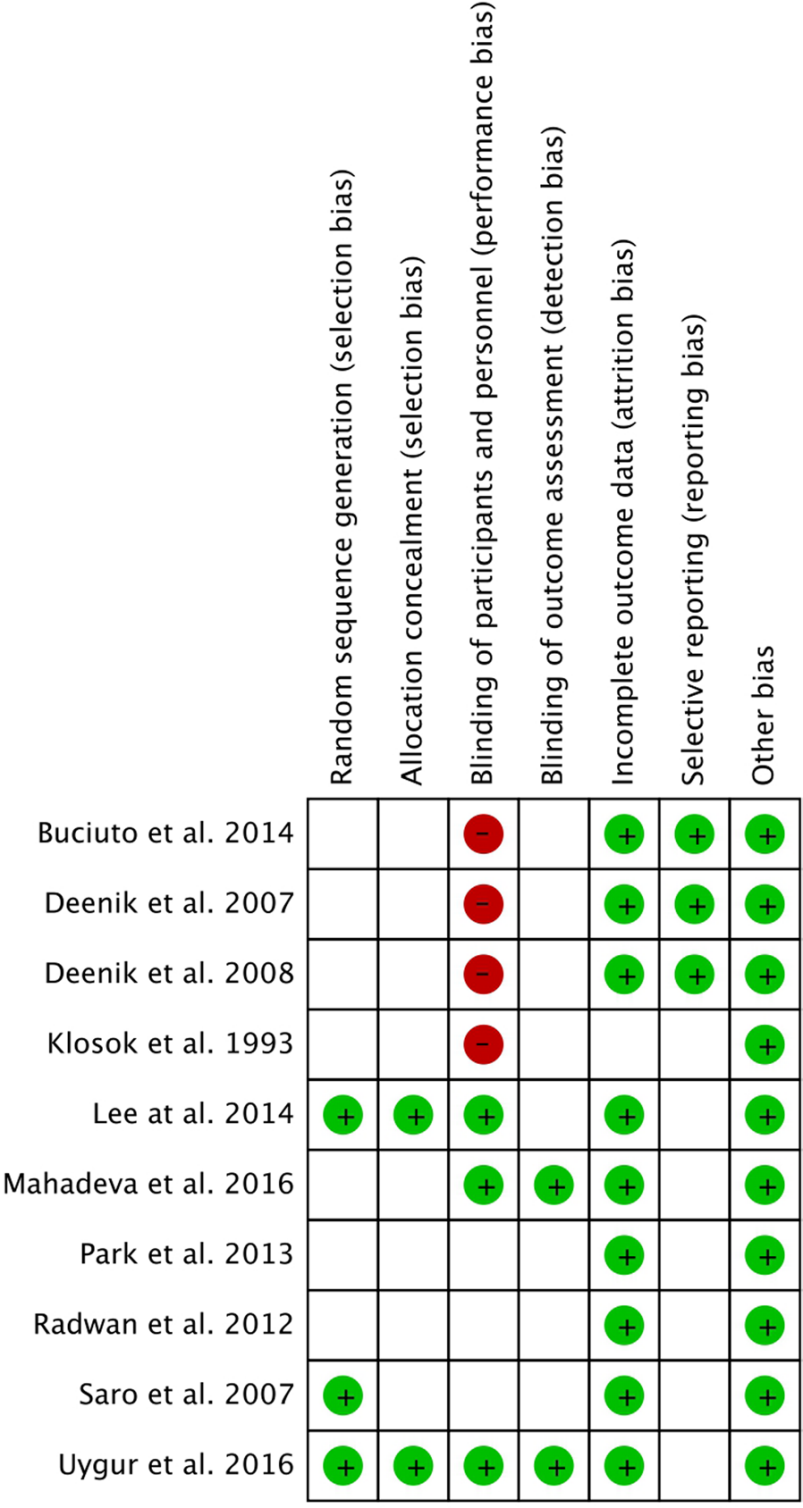
Risk of bias summary (Green: low risk; white: unknown risk; red: High risk)

The quality of RCTs was evaluated in accordance with Review Manager (RevMan) version 5.3 (The Nordic Cochrane Centre, The Cochrane Collaboration, Copenhagen, 2014) software to assess for the risk of bias. If there was a conflict between the two reviewers, a third reviewer was consulted to arrive at a decision. The evaluation methods consisted of the following steps: random sequence generation, allocation concealment, blinding, incomplete outcome data, and selective outcome reporting (Fig. 2).

### Statistical analysis

The meta-analysis was performed using the Review Manager 5.4 software package provided by the Cochrane Collaboration. For dichotomous variables, odds ratios with a confidence interval (CI) of 95% were calculated. The weighted mean difference (WMD) and the 95% CI were calculated for the continuous variables. Heterogeneity was checked with both the chi^2^ and the *I*^2^ test. *I*^2^ varies from 0 to 100%, considering the values of 25, 50 and 75% as low, moderate and high heterogeneity, respectively. A fixed effects model was adopted if there was no statistical evidence of heterogeneity, and a random effects model was adopted if significant heterogeneity was observed. Publication bias was evaluated using the funnel plot diagrams.

## Results

### Baseline data

The main characteristics of the 10 included studies are shown in Table 1 [5–14].The sample sizes ranged from 60 to 136 patients, with a total of 985 patients. The average age of the patients varied from 32.7 to 54 years. Stratification by gender was reported in eight studies, with a percentage of males ranging from 0 to 13.7%. The patients had a follow-up between 12 and 56.4 months.

**Table 1:** Characteristics of randomized clinical trials included in the meta-analysis

Studies	Arms		n			% Men	Patients selection	Mean age		Follow-up (months)
	1	2	Arm 1	Arm 2	Total			Arm 1	Arm 2	
Deenik A. et al. 2008 [7]	Chevron distal	Scarf	70	66	136	ns	IMA: mild < 11°, moderate 11°–17°, severe > 17°	ns	ns	31.2
Deenik A.R. et al. 2007 [6]	Chevron distal	Scarf	47	49	96	ns	-	43	45	27
Mahadevan et al. 2016 [10]	Chevron + Plantar extension	Scarf	60	49	109	10.71	IMA: 10°–21°	50.7	50.7	12
Radwan Y.A. et al. 2012 [12]	Chevron distal	Percutaneous distal metatarsal osteotomy	31	29	60	11.67	HVA: > 37°	35.7	32.7	19.5
							IMA: > 20°			
Uygur et al. 2016 [14]	Chevron distal	Lindgren-Turan	34	32	66	0	HVA: 20°–40°	45.8	46.9	26.1
							IMA: 14°–20°			
Klosok J.K. et al. 1993 [8]	Chevron distal	Wilson	45	42	87	13.73	-	45	45	38
Saro C. et al. 2007 [13]	Chevron distal	Lindgren	49	50	99	6	HVA: 20°–44°	48	48	56.4
							IMA: > 20°			
Buciuto R. et al. 2014 [5]	Chevron distal	Mitchell	60	60	120	0	HVA: mild < 30°, moderate < 40°	50	54	36
							IMA: mild < 13° moderate > 13°			
Lee K.B. et al. 2014 [9]	Chevron distal	Chevron proximal	46	46	92	0	HVA: ≥ 20°	53.8	53.8	ns
							IMA: ≥ 14°			
Park H.W. et al. 2013 [11]	Chevron distal	Chevron proximal	54	56	110	0	HVA: ≥ 40°	53	54	39
							IMA: ≥ 17°			

### Radiological outcomes

The hallux valgus angle (HVA) was described in 11 studies. Four were excluded—one for not providing the standard deviation and three for being isolated non-comparable studies. Seven studies were included: three related to the scarf technique, with 341 patients; two studies compared distal Chevron osteotomy with the Lindgren technique, with 156 patients; and two studies comparing proximal and distal chevron osteotomies, with 202 patients.

**Fig. 3:**
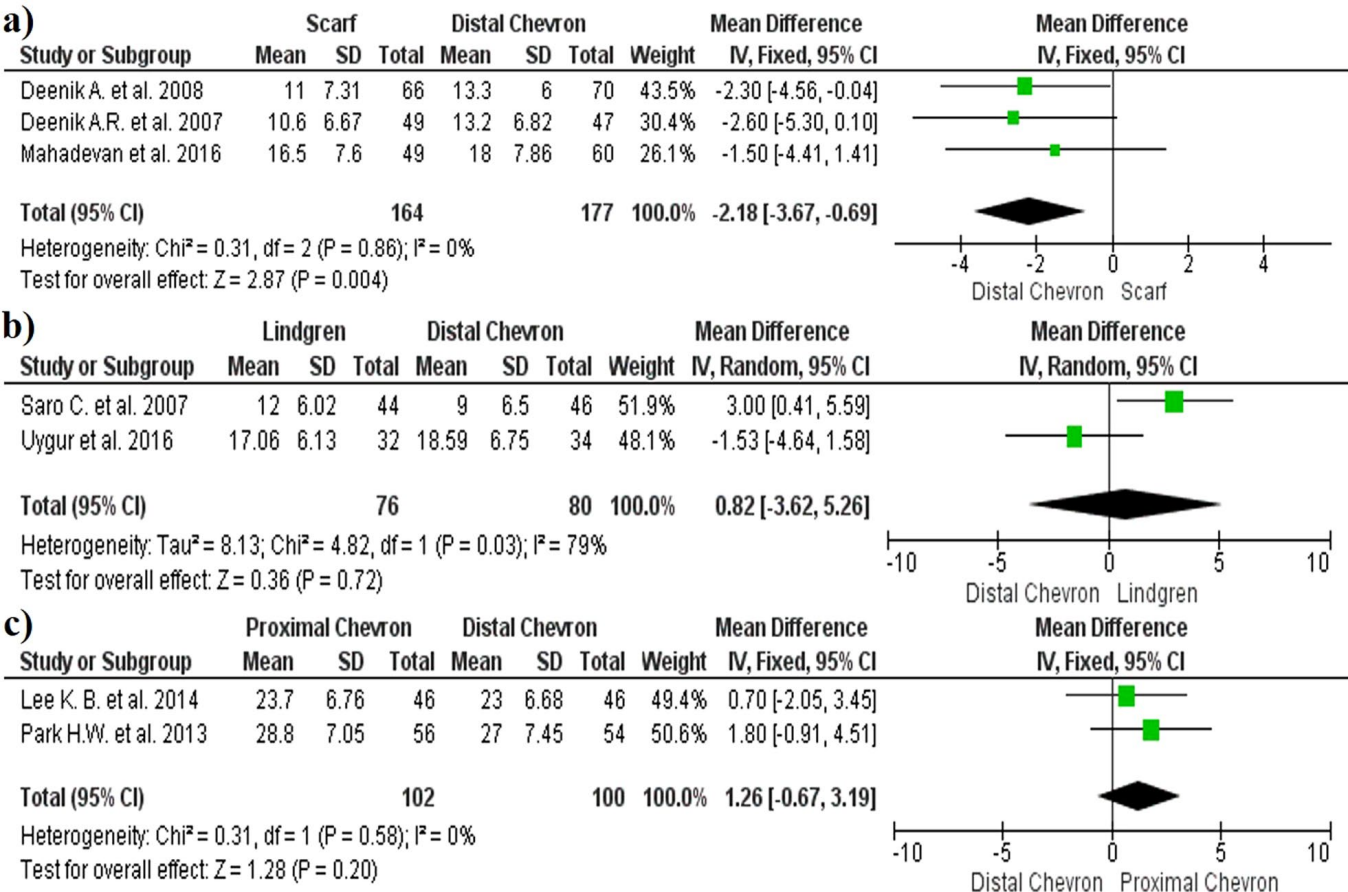
Forest plot of Hallux Valgus Angle: **a** HVA correction between distal chevron and scarf; **b** HVA correction between distal chevron and Lindgren; **c** HVA correction between distal chevron and proximal chevron

**Fig. 4:**
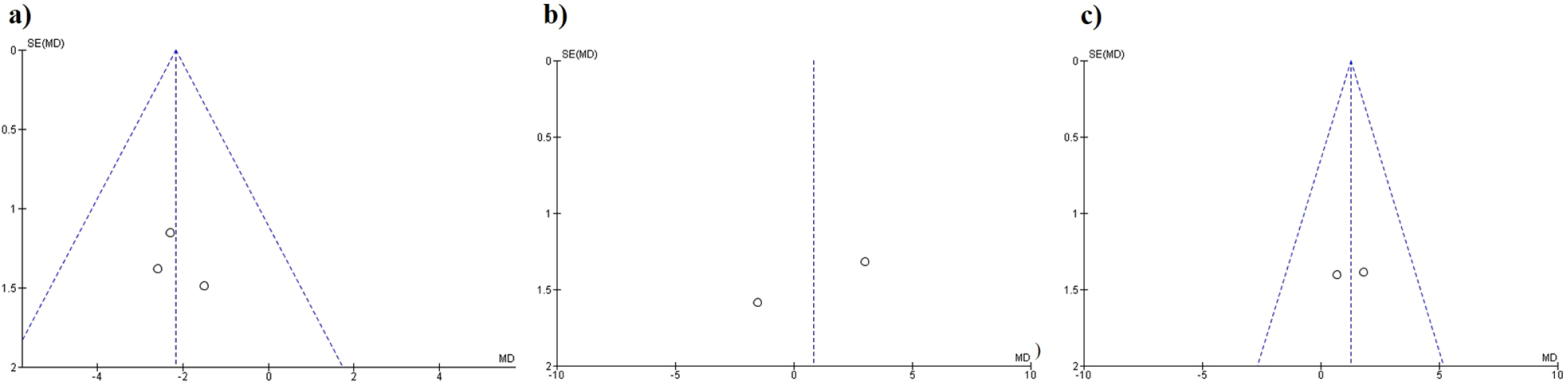
Hallux Valgus Angle funnel plot diagrams: **a** Distal chevron versus scarf; **b** Distal chevron versus Lindgren; **c** Distal chevron versus proximal chevron

The results regarding the change between HVA showed a greater reduction in the distal chevron osteotomy when compared with the scarf technique (MD − 2.18, 95% CI − 3.67, − 0.69, *p* = 0.004, *I*^2^ = 0%) (Fig. 3a). There were no significant differences in HVA when the Lindgren and distal chevron techniques were compared (MD 0.82, 95% CI − 3.62, 5.26, *p* = 0.72, *I*^2^ = 79%) (Fig. 3b). No significant differences were observed between the proximal and distal chevron osteotomies (MD 1.26, 95% CI − 0.67, 3.19, *p* = 0.2, *I*^2^ = 0%) (Fig. 3c). The funnel plot diagrams did not show the existence of publication bias (Fig. 4).

The intermetatarsal angle (IMA) was described in 10 studies, of which three were excluded because they were isolated, non-comparable studies. Seven studies were included: three, with a total of 341 patients, dealt with the scarf technique; two studies, with a total of 156 patients, compared distal Chevron osteotomy with the Lindgren technique; and two studies compared the proximal and distal chevron osteotomies.

**Fig. 5:**
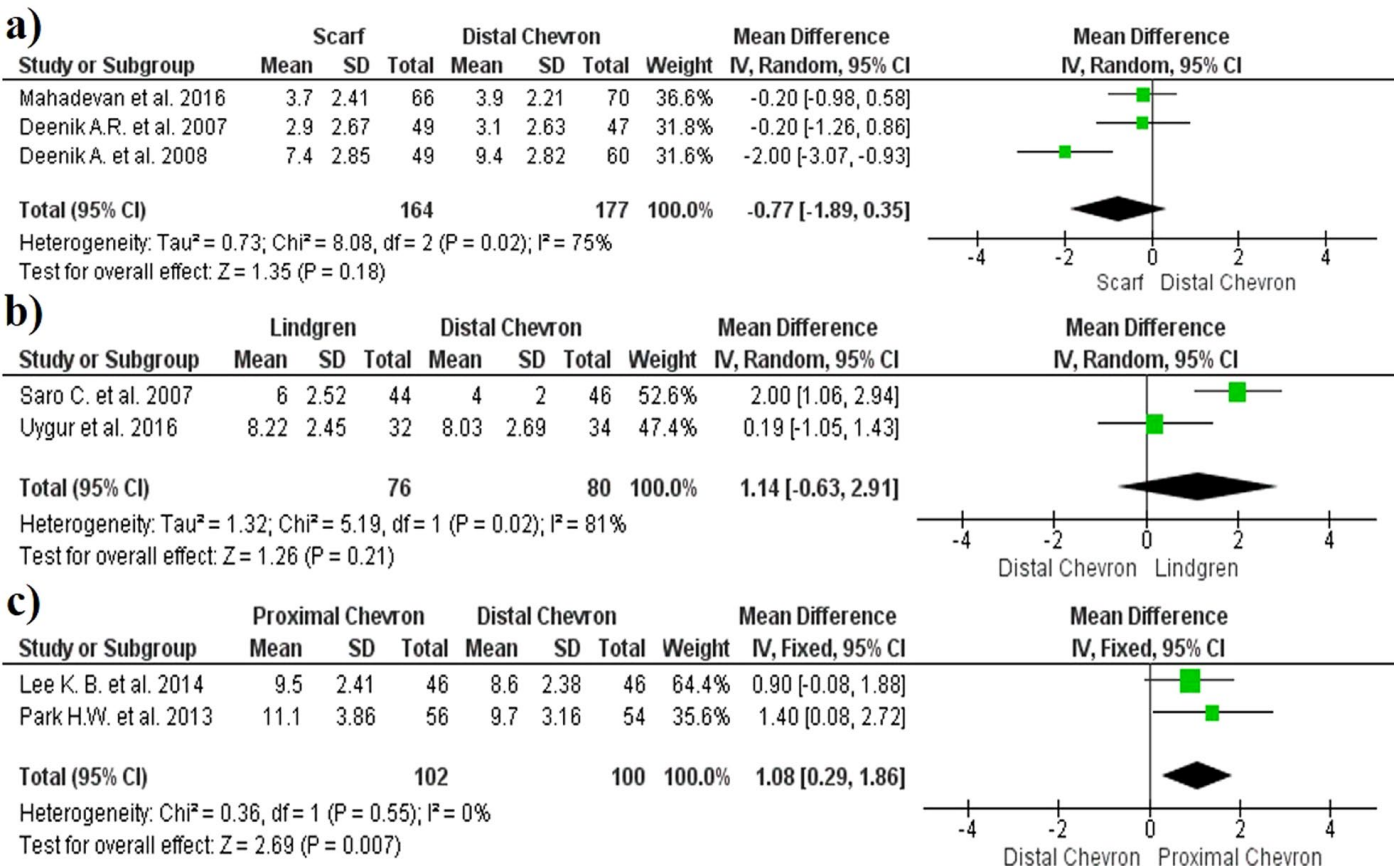
Forest plot of Inter Metatarsal Angle: **a** IMA correction between distal chevron and scarf; **b** IMA correction between distal chevron and Lindgren; **c** IMA correction between distal chevron and proximal chevron

**Fig. 6:**
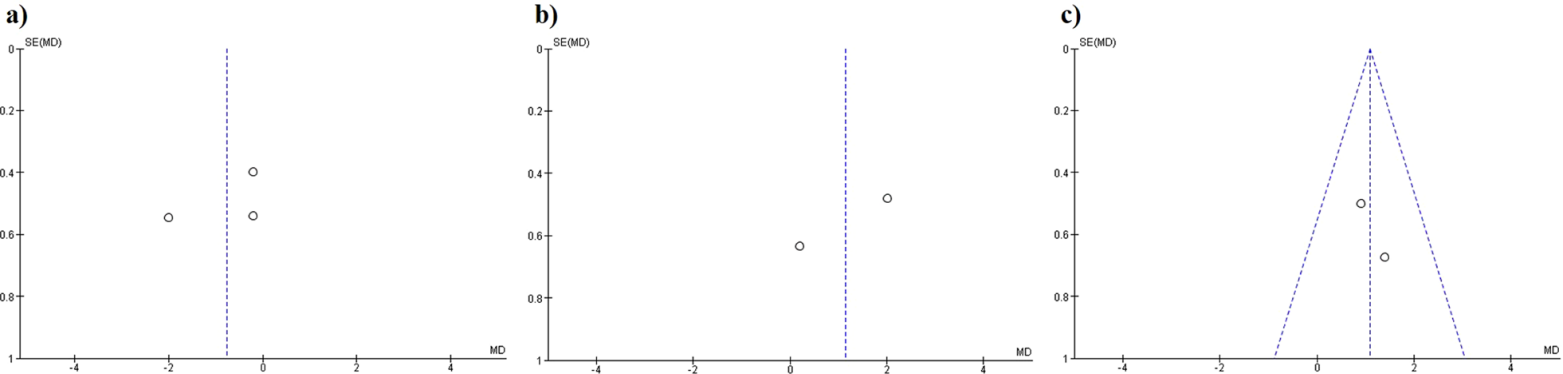
Inter Metatarsal Angle funnel plot diagrams: **a** Distal chevron versus scarf; **b** Distal chevron versus Lindgren; **c** Distal chevron versus proximal chevron

The results regarding pre- and postoperative IMA values did not show significant differences between the distal chevron and scarf osteotomies (MD − 0.77, 95% CI 1.89, 0.35, *p* = 0.18, *I*^2^ = 75%) (Fig. 5a). No significant differences were observed between the Lindgren and distal chevron techniques (MD 1.14, 95% CI − 0.63, 2.91, *p* = 0.21, *I*^2^ = 81%) (Fig. 5b). Finally, a greater reduction in the IMA was observed in proximal chevron than in distal chevron osteotomies (MD − 1.08, 95% CI 0.29, 1.86, *p* = 0.007, *I*^2^ = 0%) (Fig. 5c). The funnel plot diagrams did not show the existence of publication bias (Fig. 6).

### Clinical outcomes

The AOFAS scale was described in seven studies. Three studies were excluded, one due to lack of information on the standard deviation and two because they were isolated non-comparable studies. Four studies were included. Two studies, with a total of 156 patients, compared distal chevron osteotomy with the Lindgren technique, and two studies, with a total of 202 patients, compared distal chevron with proximal chevron osteotomy.

**Fig. 7:**
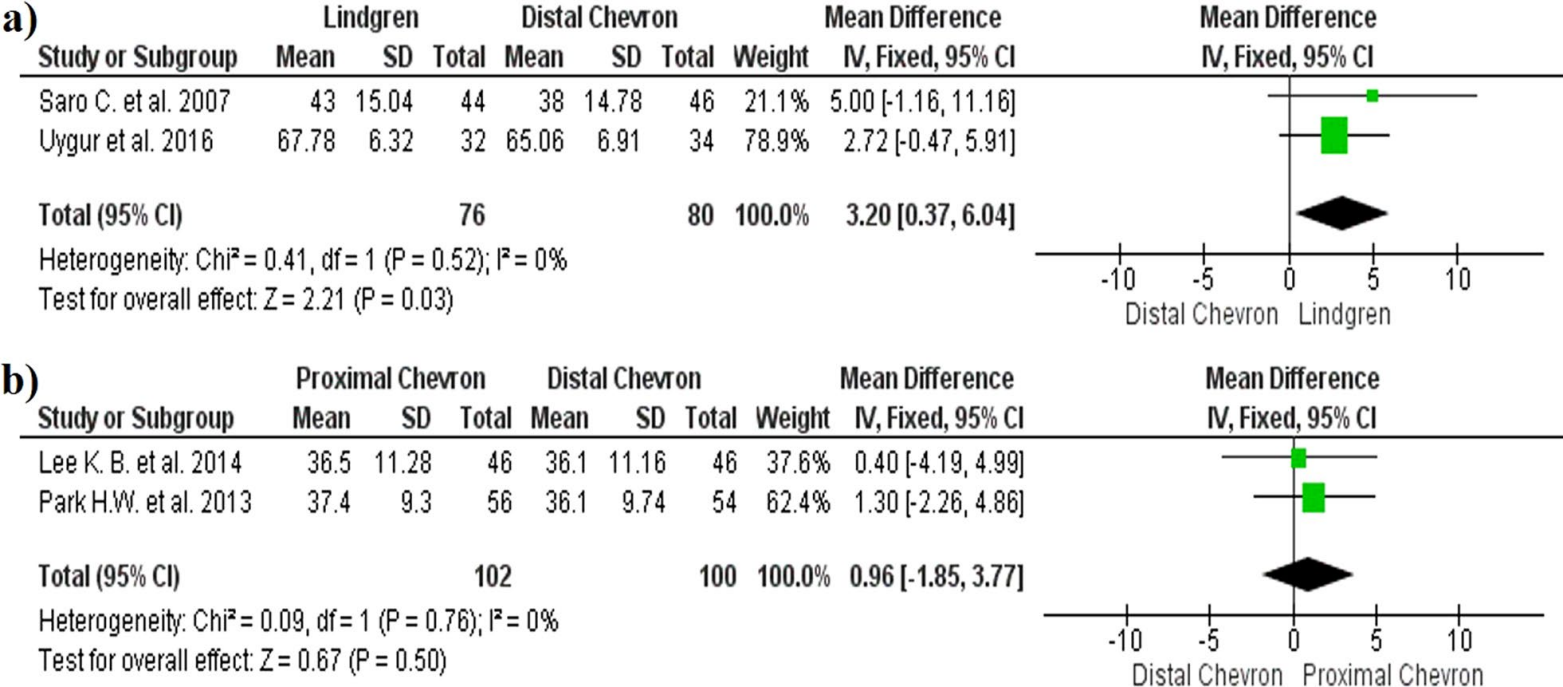
Forest plot of AOFAS scale: **a** AOFAS correction between distal chevron and Lindgren; **b** AOFAS correction between distal chevron and proximal chevron

**Fig. 8:**
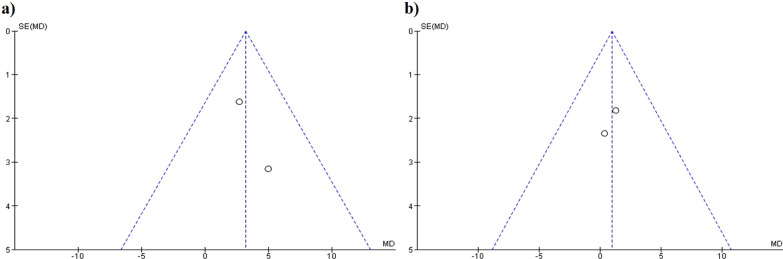
AOFAS funnel plot diagrams: **a** Distal chevron versus Lindgren; **b** Distal chevron versus proximal chevron

The AOFAS before and after surgery showed a significantly improvement in the Lindgren technique compared with distal chevron (MD 3.20, 95% CI: 0.37, 6.04, *p* = 0.03, *I*^2^ = 0%) (Fig. 7a). No significant differences were observed between the proximal and distal chevron (MD 0.96, 95% CI − 1.85, 3.77, *p* = 0.5, *I*^2^ = 0%) (Fig. 7b). The funnel plot diagrams did not show the existence of publication bias (Fig. 8).

## Discussion

The purpose of this study was to compare the efficacy of distal chevron osteotomy to that of other operative techniques for correcting to hallux valgus in different randomized studies. We used radiological parameters (HAV and IMA) and AOFAS as a clinical measure.

Regarding the radiological results, the reduction in the HVA was higher with the distal chevron osteotomy than the scarf technique. In the three included studies, the mean difference in HVA correction was 2.18° in favor of distal chevron osteotomy. A recent randomized study comparing percutaneous distal chevron osteotomy with scarf osteotomy showed similar results, with a mean correction of 2.7° higher in distal chevron osteotomy [16]. When the distal chevron technique (spread plantarly) was compared with the scarf technique, both techniques showed similar AHV corrections with respect to our study, with 1.1° in favor of the scarf technique. There are a few studies in the literature comparing distal chevron and scarf that have a I–II evidence level. There are case series of both techniques, but all of them have a III–IV evidence level, and are therefore not comparable to this meta-analysis. A systematic review of these articles was reported in 2012, but did not include the HVA outcome [18]. Among the case series of scarf osteotomy, corrections of the HVA of 25° on average have been published, always associated with Akin phalanx osteotomy [19]. For chevron osteotomy, large HVA corrections of more than 30° have also been described [20].

In this meta-analysis, the mean correction of HVA with distal chevron was 23.22°, slightly higher than the 17.1° described by Schuh et al. in a systematic review of proximal osteotomies, including 446 proximal chevron osteotomies.

When it came to the IMA, the proximal chevron osteotomy showed a greater reduction than the distal chevron, so the proximal chevron osteotomy could be the first option if a greater radiological correction is required. In contrast, there was no difference between distal chevron osteotomy and Lingdren or scarf osteotomy. There was only one meta-analysis that compared distal chevron and scarf osteotomy, which was published by Smith et al. in 2012. This study made a compilation of 31 studies with 1351 patients. The researchers’ results showed a significant reduction of 0.88° in favor of scarf osteotomy [18]. Our study showed no significant difference in IMA correction between these two procedures. According to the literature, scarf osteotomy showed a greater correction of IMA [19].

Lateral translation in both osteotomies is limited, as bone contact is essential for adequate fixation. In X-rays, the appearance of the first metatarsal after chevron osteotomy is different from after scarf osteotomy, as the chevron osteotomy shows a valgus deformity in the long axis of the first metatarsal. In scarf osteotomy, the correction of the longitudinal axis is more aligned. However, the biomechanical axis and therefore the IMA are defined by the corrected metatarsal head and not by the shape of the axis [21]. Smith et al. measured the center of the metatarsal head separately from the magnitude of the bunionette. This method of measurement has been found to be the most reliable [22].

However, the radiological measures, both HVA and IMA, are considered to be inaccurate and only include assessments in a single plane. Thus, comparisons between studies should be evaluated carefully [23].

Regarding the AOFAS, Lingdren osteotomy could be chosen over distal chevron osteotomy based on clinical criteria. On the other hand, there is no difference between distal and proximal chevron osteotomies in the improvement of the AOFAS.

When comparing the minimally invasive technique with chevron osteotomy, no short- and long-term differences were found between quality of life scores, radiological parameters and range of motion. Only higher patient satisfaction was observed with the minimally invasive technique at 12 weeks [24, 25].

Some of the limitations of this meta-analysis were the sample sizes of the different studies, so there was no subgroup analysis. Regarding quality of life or pain outcomes, only the AOFAS and VAS could be compared. Also, the traditional standard chevron procedure is used for moderate hallux valgus correction. However, in recent years, due to the continuous improvement of the surgical method, it has also been applied to severe hallux valgus. The Chevron surgical approach is under continuous development.

## Conclusion

In conclusion, this meta-analysis considered articles not included in recent meta-analyses. It is also the only meta-analysis to our knowledge that includes an analysis of the publication bias (funnel plot) of each outcome. According to the results of this meta-analysis, Lingdren osteotomy with respect to produces a greater clinical improvement than distal chevron osteotomy, as assessed by the AOFAS scale. Similarly, distal chevron osteotomy produces better clinical results than the scarf technique. Finally, proximal chevron osteotomy achieves a greater reduction in IMA than distal chevron osteotomy.

## Abbreviations

AOFAS: American Orthopaedic Foot & Ankle Society Score
HV: Hallux Valgus
HVA: Hallux Metatarsal Phalangeal Angle
IMA: Intermetatarsal Angle
MTP: Metatarsophalangeal joint
PRISMA: Preferred Reporting Items for Systematic Reviews and Meta-Analyses
WMD: Weighted Mean Difference
